# Induction of Cytotoxic T-Lymphocyte Responses Upon Subcutaneous Administration of a Subunit Vaccine Adjuvanted With an Emulsion Containing the Toll-Like Receptor 3 Ligand Poly(I:C)

**DOI:** 10.3389/fimmu.2018.00898

**Published:** 2018-04-30

**Authors:** Signe Tandrup Schmidt, Gabriel Kristian Pedersen, Malene Aaby Neustrup, Karen Smith Korsholm, Thomas Rades, Peter Andersen, Camilla Foged, Dennis Christensen

**Affiliations:** ^1^Department of Pharmacy, Faculty of Health and Medical Sciences, University of Copenhagen, Copenhagen, Denmark; ^2^Department of Infectious Disease Immunology, Statens Serum Institut, Copenhagen, Denmark

**Keywords:** subunit vaccine, adjuvant, nanoemulsion, cytotoxic T-lymphocytes, formulation, poly(I:C)

## Abstract

There is an unmet medical need for new subunit vaccines that induce cytotoxic T-lymphocyte (CTL) responses to prevent infection with a number of pathogens. However, stimulation of CTL responses *via* clinically acceptable subcutaneous (s.c.) and intramuscular (i.m.) injection is challenging. Recently, we designed a liposomal adjuvant [cationic adjuvant formulation (CAF)09] composed of the cationic lipid dimethyldioctadecylammonium (DDA) bromide, a synthetic monomycoloyl glycerol analog and polyinosinic:polycytidylic acid, which induce strong CTL responses to peptide and protein antigens after intraperitoneal administration. By contrast, CAF09 does not stimulate CTL responses upon s.c. or i.m. injection because the vaccine forms a depot that remains at the injection site. Hence, we engineered a series of nanoemulsions (CAF24a–c) based on the active components of CAF09. The oil phase consisted of biodegradable squalane, and the surface charge was varied systematically by replacing DDA with zwitterionic distearoylphosphoethanolamine. We hypothesized that the nanoemulsions drain to the lymph nodes to a larger extent than CAF09, upon s.c. co-administration with the model antigen chicken egg ovalbumin (OVA). This results in an increased dose fraction that reaches the draining lymph nodes (dLNs) and subsequently activates cross-presenting dendritic cells (DCs), which can prime CTL responses. Indeed, the nanoemulsions induced antigen-specific CD8^+^ T-cell responses, which were significantly higher than those stimulated by OVA adjuvanted with CAF09. We explain this by the observed rapid localization of CAF24a in the dLNs and the subsequent association with conventional DCs, which promotes induction of CTL responses. Uptake of CAF24a was not specific for DCs, because CAF24a was also detected with B cells and macrophages. No measurable dose fraction of CAF09 was detected in the dLNs within the study period, and CAF09 formed a depot at the site of injection. Importantly, s.c. vaccination with OVA adjuvanted with CAF24a induced significant levels of specific lysis of antigen-pulsed splenocytes were induced after, which was not observed for OVA adjuvanted with CAF09. Thus, CAF24a is a promising adjuvant for induction of CTL responses upon s.c. and i.m. immunization, and it offers interesting perspectives for the design of vaccines against pathogens for which CTL responses are required to prevent infection.

## Introduction

Vaccine-mediated induction of antigen-specific cytotoxic T-lymphocyte (CTL) responses is considered essential for preventing and/or treating a number of intracellular infections and cancers (1, 2). Stimulation of CTL responses against exogenously derived vaccine antigens remains a challenge, although a number of recent studies suggest that this can be achieved by activating specific innate immune pathways (3, 4). Specialized CD8α^+^ and CD103^+^ dendritic cell (DC) subsets are capable of priming CTL responses *via* the so-called cross-priming pathway (1, 2, 5). Cross-presenting DCs can present peptides from exogenously derived antigens on major histocompatibility complex (MHC)-I, which in combination with co-stimulatory signals prime antigen-specific CD8^+^ T cells that subsequently differentiate into CTLs (2). It is generally recognized that CD8α^+^ DCs reside in the lymph nodes (LNs), whereas CD103^+^ DCs primarily localize to mucosal sites and the skin, from where they migrate to the draining lymph nodes (dLNs) upon activation (6). Cross-presentation of vaccine antigens by DCs can be enhanced by simultaneous activation of toll-like receptor (TLR)3, which is expressed at high levels in the endosomes of cross-priming DCs. Double-stranded RNA binds and activates TLR3, which triggers intracellular signaling cascades and ultimately upregulates the expression of type I interferons (IFNs) (7, 8). Thus, targeting of vaccines to LN-resident, CD8α^+^ DCs upon subcutaneous (s.c.) and intramuscular (i.m.) immunization is a promising strategy for induction of strong, systemic CTL responses.

Recently, we designed a liposomal cationic adjuvant formulation (CAF)09, composed of dimethyldioctadecylammonium (DDA) bromide, the synthetic monomycoloyl glycerol (MMG) analog MMG-1, and the synthetic TLR3 agonist polyinosinic:polycytidylic acid [poly(I:C)] electrostatically adsorbed to the DDA headgroups, which induces remarkably strong antigen-specific CD8^+^ T-cell responses upon co-administration with antigen by intraperitoneal (i.p.) injection (9). By contrast, CAF09-adjuvanted antigen induces only very weak CD8^+^ T-cell responses upon s.c. and i.m. immunization (9). This is likely a consequence of the vaccine forming a depot at the site of injection (SOI) (10), caused by interaction between the cationic adjuvant and interstitial proteins, eventually leading to aggregation and depot formation (11). Depot formation has been shown to prevent free drainage of the vaccine from the SOI *via* the lymphatics to the dLNs, which is a prerequisite for targeting cross-priming CD8α^+^ DCs localized in the dLNs and the spleen (5). The depot-forming ability has been shown to depend mainly on two physicochemical properties of liposomal adjuvants, i.e., the surface charge and the lipid bilayer fluidity (11, 12). In addition, the size of particle-based vaccines influences the drainage properties, and nanoparticles with diameters between approximately 40 and 200 nm have been shown to drain more efficiently from the SOI to the dLNs than larger-sized nanoparticles (5, 13), because they can enter the lymphatics *via* gaps in the vascular endothelium of the lymphatic ducts (14).

Hence, we hypothesized that nanoemulsion adjuvants induce CD8^+^ T-cell responses upon s.c. immunization, because oil-in-water (o/w) emulsions have fast drainage kinetics due to their oily nature and relatively small droplet size (less than approximately 200 nm). For example, the squalene-based emulsion MF59^®^ has been shown to be cleared rapidly from the SOI by free drainage and/or uptake and transport by neutrophils (15, 16). The dose fraction reaching the dLNs by free drainage may thus be enhanced by reducing the average size of the oil droplets below 200 nm, e.g., by high-pressure homogenization or sonication (17). Examples of marketed emulsion-based vaccine adjuvants are the o/w emulsions, MF59^®^ and AS03^®^, which contains biocompatible squalene as the oil phase, and they have been reported to induce mainly humoral immune responses (17–20).

In a recent study, we designed nanoemulsions composed of unsaturated squalene, or the saturated analog squalene, as the oil phase, which was emulsified with surfactant mixtures containing four different components (i) polysorbates (Tween^®^) and sorbitans (Span^®^), (ii) the saturated cationic lipid DDA or its unsaturated analog dioleoyldimethylammonium chloride, (iii) the saturated zwitterionic distearoylphosphoethanolamine (DSPE) or its unsaturated analog dioleoylphosphoethanolamine, and (iv) the immunostimulatory lipid MMG-1. We measured antigen-specific CD4^+^ T-cell responses for the positively charged, unsaturated formulations upon s.c. immunization with the *Chlamydia trachomatis* major outer membrane protein-based fusion antigen CTH522 (21–23).

The aim of this study was to design and characterize novel nanoemulsion formulations (CAF24a–c) based on our recently optimized cationic emulsion (21) and the immunostimulatory components of CAF09. Furthermore, the adjuvanticity of CAF24a–c was compared to that of CAF09 in mice. Poly(I:C) was incorporated into the nanoemulsions, and their ability to induce CD8^+^ T-cell responses upon s.c. immunization was investigated. We hypothesized that nanoemulsions have potential as CTL-inducing adjuvants, when combined with immunostimulators promoting activation of cross-priming DCs, which in this case is a combination of MMG-1 and poly(I:C). We show that CAF24a drains to the dLNs a few hours after i.m. immunization and induces significantly higher and functional antigen-specific CTL responses compared to liposome-based CAF09.

## Materials and Methods

### Materials

Squalane, Tween^®^ 60, Span^®^ 60 and γ-irradiated poly(I:C) were obtained from Sigma-Aldrich (St. Louis, MO, USA). DDA and MMG-1 (24) were purchased from NCK A/S (Farum, Denmark), DSPE was obtained from Avanti Polar Lipids (Alabaster, AL, USA), and endotoxin-free ovalbumin (OVA) was acquired from Hyglos GmbH (Bernried am Starnberger See, Germany). AlexaFluor 647-conjugated OVA was from ThermoFisher Scientific Inc. (Waltham, MA, USA), and 3,3′-dioctadecyloxacarbocyanine perchlorate (DiO) was provided by Life Technologies (Nærum, Denmark). All other chemicals were purchased from commercial suppliers and used at analytical grade.

### Preparation of Emulsions

Weighed amounts of MMG-1, DDA, and DSPE, respectively, were dissolved in 99% (v/v) EtOH and mixed in glass vials (Table 1). The surfactant mixtures were dried under a gentle N_2_ stream for 2 h followed by air-drying overnight to remove trace amounts of solvent eventually resulting in the formation of a lipid film. Span^®^ 60 was weighed into the dry surfactant mixture, because it is poorly soluble in EtOH. The oil phase was weighed into the vial and heated to 60°C for 10 min with intermittent vortex mixing to melt the surfactants and the oil phase. Poly(I:C) dissolved in water-for-injection (10 mg/ml) was added to the oil phase, and the mixture was heated to 60°C for 10 min with intermittent vortex mixing to form a water-in-oil (w/o)-emulsion. The second water phase, consisting of Tween^®^ 60 dissolved in Tris-buffer (10 mM, pH 7.4), was added to the w/o emulsion. A pre-emulsion was prepared by high shear mixing (HSM) using a Heidolph Silent Crusher equipped with a 6F shearing tool (Heidolph Instruments GmbH, Schwabach, Germany) at 60°C and 26,000 rpm for 5 min, and it was subsequently microfluidized six times at 20,000 psi using a LV1 Low Volume Homogenizer (Microfluidics, Westwood, MA, USA). Fluorescently labeled CAF24a used for *in vivo* tracking studies was prepared by addition of DiO dissolved in EtOH during the preparation of the lipid film, resulting in a concentration of 0.03 mg/ml in the final emulsion.

**Table 1:** The composition of the emulsions CAF24a–c with different ratios of DDA and DSPE.

Emulsion	CAF24a	CAF24b	CAF24c
Squalane	10,000	10,000	10,000
Span^®^ 60	960	960	960
Tween^®^ 60	1,040	1,040	1,040
Monomycoloyl glycerol	100	100	100
Poly(I:C)	50	50	50
DDA	200	100	0
DSPE	0	119	237

*The values indicate the dose of each component in microgram administered in a volume of 100 µl*.

*CAF, cationic adjuvant formulation; DDA, dimethyldioctadecylammonium; DSPE, distearoylphosphoethanolamine*.

### Preparation of CAF09

Weighed amounts of DDA and MMG-1 were dissolved in 99% (v/v) EtOH and mixed in a glass vial at an 82:18 M ratio. The lipid mixture was dried under a gentle N_2_ stream for 2 h followed by air-drying overnight to remove trace amounts of solvent. The lipid film was rehydrated in Tris-buffer (10 mM, pH 7.4) by HSM by using a Heidolph Silent Crusher equipped with a 6F shearing tool at 60°C and 26,000 rpm for 15 min. Subsequently, poly(I:C) was added slowly to the liposomes with concomitant HSM at 60°C. The final concentrations in the resulting dispersion were 2.5/0.5/0.5 mg/ml DDA/MMG-1/poly(I:C). Fluorescently labeled CAF09 used for *in vivo* tracking studies was prepared by addition of DiO dissolved in EtOH during the preparation of the lipid film, resulting in a concentration of 0.03 mg/ml in the final formulation.

### Physicochemical Characterization

The intensity-weighted average hydrodynamic diameter (*z*-average) and polydispersity index (PDI) of the emulsions and the liposome dispersions were determined by dynamic light scattering using photon correlation spectroscopy. Undiluted emulsion samples or liposome samples diluted 10 times were analyzed at 25°C using a Zetasizer Nano ZS (Malvern Instruments Ltd., Worcestershire, UK) equipped with a 633 nm laser and 173° detection optics. For undiluted samples, the particle sizes were independent of the sample concentration. For viscosity and refractive indexes, the values of water were used (values provided by Malvern Instruments). The particle size distribution was reflected in the PDI, which ranges from 0 for a monodisperse to 1.0 for an entirely heterodisperse dispersion. The zeta-potentials of the formulations were measured for samples diluted 100 times in milliQ-water. Zetasizer Software v7.11 (Malvern Instruments Ltd.) was used for acquisition and analysis of the data. The morphology of the emulsions with or without poly(I:C) was investigated by cryo-transmission electron microscopy (cryo-TEM) using a Tecnai G2 20 TWIN transmission electron microscope (FEI, Hillsboro, OR, USA) mounted with a 4 × 4 K charged-coupled device Eagle camera from FEI, essentially as described elsewhere (25).

### Immunization of Mice

All animal experiments were conducted in accordance with the national Danish guidelines for animal experiments and EU directive 2010/63/EU for animal experiments, as approved by the Danish Animal Experiments Inspectorate (license number 2014-15-2934-01065). All procedures were refined to provide maximal comfort and minimal stress for the animals. Female, 6- to 8-week-old C57BL/6 mice were purchased from Harlan (Horst, Netherlands) and allowed free access to food and water. The vaccines were prepared by mixing OVA with adjuvant 30 min prior to administration. For *in vivo* tracking studies, mice were immunized once i.m. with 10 µg AF647-OVA, AF647-OVA/DiO-CAF09, and AF647-OVA/DiO-CAF24a, respectively, in each quadriceps. Mice dosed i.m. with 50 µl isotonic Tris-buffer served as negative controls. For immunogenicity and *in vivo* target cell lysis studies, mice were immunized three times at 2-week intervals s.c. at the base of the tail or i.p. as stated, with 1 or 10 µg OVA, respectively, in a dose volume of 100 µl. The dose of CAF24a–c is given in Table 1. For CAF09, the dose was 250/50/50 μg DDA/MMG-1/poly(I:C). Unadjuvanted OVA was used as a negative control.

### Preparation of Organs

The biodistribution of fluorescently labeled vaccines was evaluated 1, 5, 18, 24, 48, and 120 h after immunization, whereas immune responses and antigen-specific lysis of target cells were evaluated 1 week after the final immunization. The spleens, the inguinal LNs (to which vaccines administered s.c. and i.m. are draining), the mediastinal and tracheobronchial LNs (to which vaccines administered i.p. are draining), and blood were harvested. The quadriceps muscles were also harvested for the *in vivo* tracking studies. Single cell suspensions were generated from the spleens and LNs by passing the organs through a nylon mesh cell-strainer followed by washes with phosphate-buffered saline (PBS, pH 7.4) and RPMI 1640 (Invitrogen, Carlsbad, CA, USA). The muscles were treated with enzymes A, D, and P of the Skeletal Muscle Dissociation Kit (Miltenyi Biotec GmbH, Bergisch Gladbach, Germany) according to the manufacturer’s instructions. Cells from organs were resuspended in RPMI 1640 supplemented with 10% (v/v) heat-inactivated fetal calf serum, 5 × 10^−6^ M β-mercaptoethanol, 1% (v/v) penicillin–streptomycin, 1% (v/v) sodium pyruvate, 1 mM l-glutamine, and 10 mM HEPES (cRPMI), as described elsewhere (24). Blood was harvested in EDTA-tubes, and lymphocytes were isolated by placing the blood on Lympholyte (Cedarlane, Burlington, CA, USA) followed by centrifugation at 900 *g* for 20 min. The lymphocytes were subsequently washed with PBS and resuspended in FACS-buffer [PBS with 1% (v/v) fetal calf serum].

### Pentamer Flow Cytometry

The frequency of antigen-specific CD8^+^ T cells in the blood was determined by pentamer flow cytometry. Briefly, 10^6^ cells/well were stained with H2-K^b^-SIINFEKL:PE (ProImmune, Oxford, UK), anti-mouse CD19:PerCP-Cy5.5 antibody (Ab, 1D3) and CD4:eFluor780 Ab (RM4-5) from eBiosciences (San Diego, CA, USA), and CD8:BV421 Ab (53-6.7), CD44:APC Ab (IM-7), and CD62L:FITC Ab (MEL-14), all from BD Biosciences (San Jose, CA, USA). Data were acquired by using a BD Fortessa flow cytometer (BD Biosciences) and analyzed by using the FlowJo vX software (Tree Star, Ashland, OR, USA) identifying immune cell subsets (Figure S1 in Supplementary Material).

### Evaluation of Cellular Association With Fluorescently Labeled Vaccines

Single cell suspensions of LNs and quadriceps from mice immunized i.m. with fluorescently labeled antigen and adjuvants were surface stained with anti-mouse Ly6G-PE Ab (1A8), CD11b:PerCp-Cy5.5 Ab (M1/70), CD11c:BV421 Ab (HL3), I-A/I-E:BV605 Ab (M5/114.15.2), and CD19:BV786 Ab (1D3) from BD Biosciences, F4/80:PE-Cy7 Ab (BM8, eBiosciences), and Ly6C:APC-Cy7 Ab (HK1.4, BioLegend, San Diego, CA, USA). The composition of the panel and the analysis of the cell subsets performed essentially as described by Calabro et al. (16). Data were acquired and analyzed as described above (Figures S2 and S3 in Supplementary Material).

### Quantification of IFN-γ

Single cell suspensions of splenocytes were restimulated with 5 µg SIINFEKL (GenScript, Piscataway, NJ, USA) in cRPMI in 96-well plates containing 2 × 10^5^ cells/well for 4 days at 37°C. cRPMI medium and Concanavalin A (Sigma-Aldrich) were used as negative and positive controls, respectively. The IFN-γ concentration in the supernatant was measured by using an enzyme-linked immunosorbent assay (ELISA) kit (BD Biosciences), as previously described (24). Briefly, MaxiSorp plates were coated with capture anti-mouse IFN-γ Ab overnight at 4°C. Following blocking with 2% (w/v) skim-milk powder dispersed in PBS, the supernatant was added to the wells at eight-time dilution in PBS. After 2 h of incubation at room temperature, biotin-conjugated anti-mouse IFN-γ detection Ab was added, followed by streptavidin-conjugated horseradish peroxidase. Detection was performed with 3,3′,5,5′-tetramethylbenzidine (Kem-En-Tec, Taastrup, Denmark), and the reaction was stopped with 0.2 M H_2_SO_4_. The optical density was read at 450 nm with 570 nm correction.

### Lysis of Target Cells *In Vivo*

Lysis of target cells *in vivo* was measured essentially as described elsewhere (26). Briefly, splenocytic single cell suspensions from naïve mice were stained with either 0.5 µM carboxyfluorescein succinimidyl ester (CFSE, Invitrogen, referred to as CFSE^lo^) or 5 µM CFSE combined with subsequent pulsing for 1.5 h with 10 µg/ml SIINFEKL peptide (referred to as CFSE^hi^). The CFSE^lo^ and CFSE^hi^ cells were mixed at a 1:1 number ratio. A total number of 1.6 × 10^6^ cells in 200 µl were injected intravenously (i.v.) into naïve mice and mice immunized with CAF24a- and CAF09-adjuvanted OVA (1 μg/dose). Mice were euthanized 24 h after injection, and the percentages of CFSE^lo^ and CFSE^hi^ cells of the total CFSE^+^ population in the spleen, dLNs, and blood were determined by flow cytometry. Specific lysis was calculated as the ratio of CFSE^hi^ to CFSE^lo^ cells in immunized mice, as compared to the average CFSE^hi^/CFSE^lo^ ratio in naïve mice (Figure S4 in Supplementary Material).

### Statistical Analysis

One-way ANOVA followed by Tukey’s multiple comparisons test was used to analyze the difference between the individual groups, and Spearman’s correlate statistics were performed using GraphPad Prism version 7.04 for Windows (GraphPad Software, La Jolla, CA, USA).

## Results

**Figure 1:**
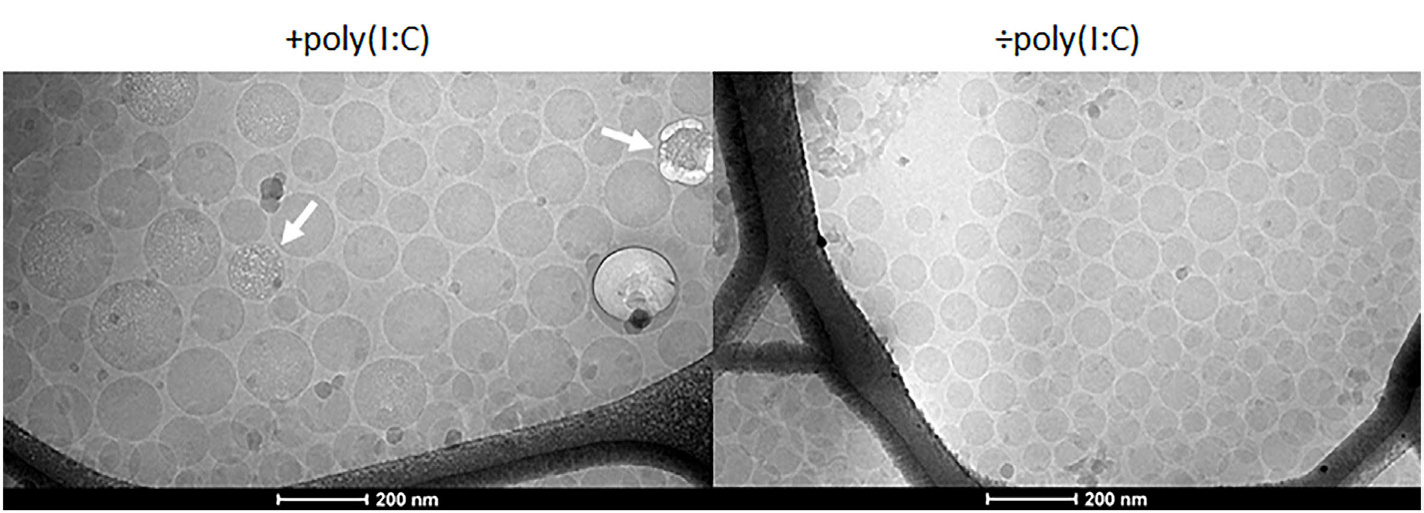
Representative cryo-transmission electron microscopy images of cationic adjuvant formulation 24a with (left) and without (right) poly(I:C). The beam damage observed in association with the emulsion droplets (arrows) may be a result of the presence of electron-dense poly(I:C).

We prepared a series of nanoemulsions based on our previously optimized emulsion (21) and the components of CAF09, and they were characterized with respect to physicochemical properties and adjuvanticity, which was evaluated in mice upon s.c. administration. The nanoemulsions were composed of the oil squalane, the surfactants Tween^®^ 60, Span^®^ 60, DDA, DSPE, and the immunostimulators MMG-1 and poly(I:C), and they were prepared by high pressure microfluidization. We included in our previously published emulsification procedure (21); (i) addition of poly(I:C) dissolved in a small volume of water-for-injection (w_1_) to the oil phase (o) and (ii) a subsequent heating step with intermittent vortexing to maximize attractive electrostatic interaction between polyanionic poly(I:C) and cationic DDA. This w_1_/o single emulsion was added to a second outer water phase (w_2_), which results in the formation of a (transient) w_1_/o/w_2_ double emulsion. The morphology of CAF24a was evaluated by cryo-TEM (Figure 1, left), which confirmed that the formulation was indeed an o/w emulsion. From the cryo-TEM pictures, it is not possible to deduce whether the resulting emulsion is a single or a double emulsion. The electron beam caused sample damage as a result of the presence of electron-dense poly(I:C) (Figure 1, left), which was not observed for a comparable and less electron-dense emulsion without poly(I:C) at identical experiment conditions (Figure 1, right). This suggests that poly(I:C) is co-localized with the oil droplets. Poly(I:C) may theoretically be localized in three different regions of the emulsion; (i) in the oil phase in a net hydrophobic complex with DDA, (ii) at the o/w interface in a net charged complex with DDA, and/or (iii) in the water phase. However, further studies are required to determine the exact spatial localization of poly(I:C) in the emulsions as a function of the poly(I:C):DDA molar ratio. This could be evaluated by using, e.g., confocal microscopy to determine the localization of fluorescently labeled poly(I:C) within the water phase and the oil phase, as previously described (25). Furthermore, the concentration of poly(I:C) in the water phase and in the oil phase may be quantified by UV spectroscopy, as previously reported (25).

High-pressure microfluidization manufacturing of the emulsions resulted in average hydrodynamic droplet diameters below approximately 200 nm (Table 2). In comparison, CAF09 prepared by HSM displayed larger particles (Table 2). As expected, replacing DDA with DSPE resulted in reduced zeta-potentials (CAF24b: −19 ± 1 mV, CAF24c: −35 ± 4 mV), while CAF09 was highly cationic (45 ± 5 mV).

**Table 2:** Average hydrodynamic diameter (*z-*average, d.nm), PDI, and zeta-potential (zp, mV) of CAF09 and CAF24a–c.

	DDA:DSPE molar ratio	*z-*average (d.nm)	PDI	Zp (mV)
CAF09	–	393 ± 39	0.21 ± 0.02	45 ± 5
CAF24a	2:0	133 ± 67	0.46 ± 0.03	28 ± 11
CAF24b	1:1	93 ± 10	0.32 ± 0.07	−19 ± 1
CAF24c	0:2	96 ± 5	0.31 ± 0.06	−35 ± 4

*n = 3, data represent mean values ± SD*.

*PDI, polydispersity index; CAF, cationic adjuvant formulation; DDA, dimethyldioctadecylammonium; DSPE, distearoylphosphoethanolamine*.

### OVA Adjuvanted With CAF24a Induces Antigen-Specific CD8^+^ T Cells

**Figure 2:**
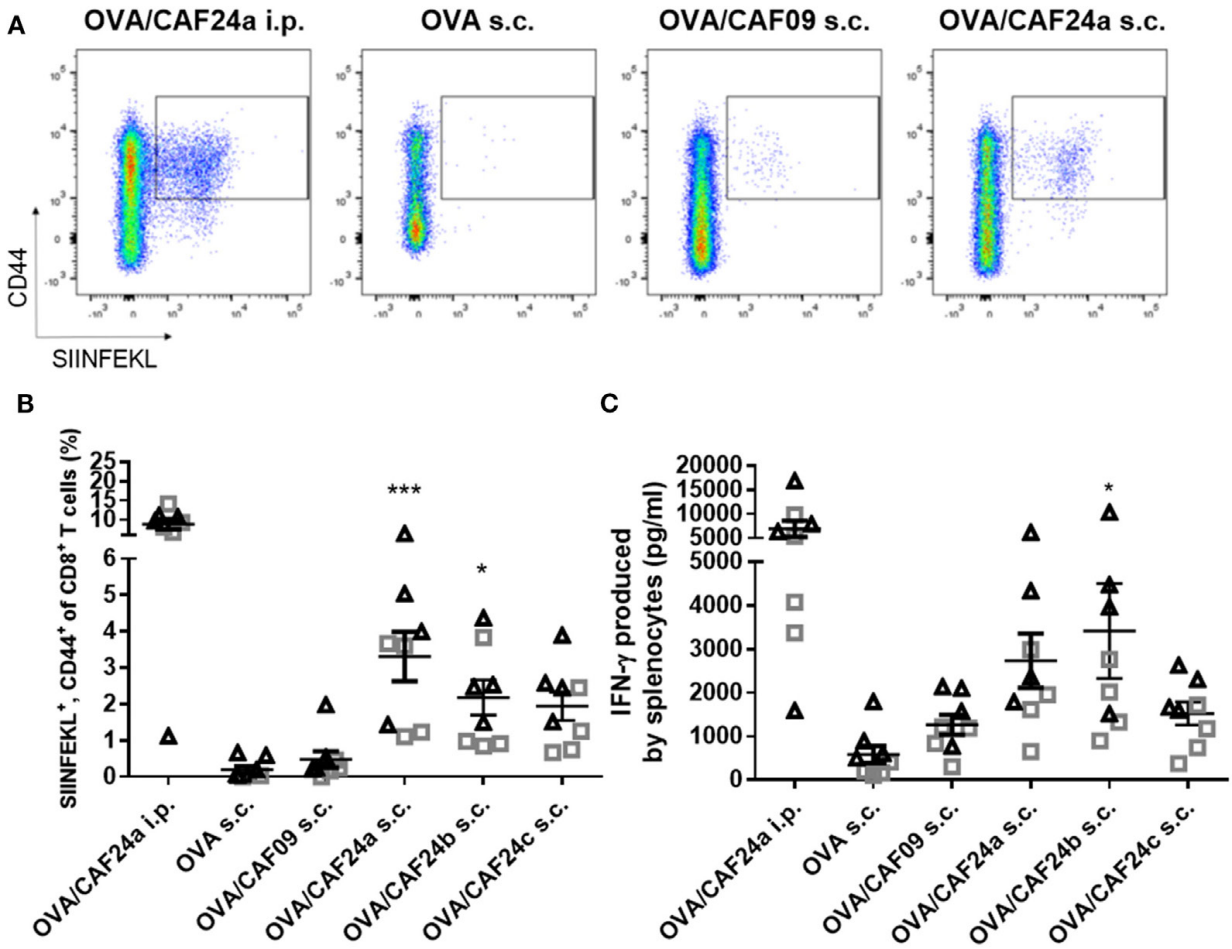
**(A)** Representative pseudocolor dot plots of activated ovalbumin (OVA)-specific CD8^+^ T cells (squared gate) induced by OVA/cationic adjuvant formulation (CAF)24a administered intraperitoneal (i.p.), and unadjuvanted OVA or OVA adjuvanted with CAF09 and CAF24a, respectively, administered subcutaneous (s.c.). **(B)** Frequency of OVA-specific CD8^+^ T cells in the blood from individual mice as determined by SIINFEKL-pentamer staining. **(C)** Secretion of interferon (IFN)-γ from splenocytes restimulated with SIINFEKL measured by using enzyme-linked immunosorbent assay (ELISA). The vaccines were administered s.c. and i.p. where indicated. *p ≤ 0.05, ***p ≤ 0.001, one-way ANOVA compared to OVA/CAF09 s.c., OVA/CAF24 i.p. was not included in the analysis, but is included for illustration. *n* = 8, data were pooled from two independent experiments (triangles and squares). Lines with bars represent mean values ± SEM.

The immunogenicity of OVA adjuvanted with CAF24a was measured upon i.p. administration and compared to the immunogenicity of OVA adjuvanted with CAF09, because we recently showed that i.p. administration of CAF09-adjuvanted OVA results in induction of remarkably strong OVA-specific CD8^+^ T-cell responses (9). Antigen-specific CD8^+^ T-cell responses in the blood were evaluated by pentamer staining for the dominant SIINFEKL CD8^+^ T-cell epitope in OVA (Figure 2A). Strong antigen-specific CD8^+^ T-cell responses were measured after i.p. immunization with CAF24a (Figure 2B), which were of comparable magnitude to the responses stimulated by CAF09-adjuvanted OVA (9). Immunization i.p. with unadjuvanted OVA was previously shown not to induce OVA-specific CD8^+^ T-cell responses (10).

The immunogenicity of the CAF24a–c-adjuvanted OVA upon s.c. immunization was compared to the immunogenicity of CAF09-adjuvanted OVA and unadjuvanted OVA (Figures 2A,B). Interestingly, CAF24a and CAF24b induced significantly stronger SIINFEKL-specific CD8^+^ T-cell responses than CAF09 after s.c. immunization (Figure 2B). In addition, OVA/CAF24c induced higher levels of antigen-specific CD8^+^ T-cell responses compared to OVA/CAF09, but the difference was not statistically significant (Figure 2B).

We measured the secretion of IFN-γ from splenocytes restimulated with SIINFEKL by ELISA. Data showed that i.p. immunization with OVA/CAF24a resulted in strong antigen-specific IFN-γ responses in the spleen (Figure 2C). For the s.c. immunized mice, OVA/CAF24b induced significantly higher IFN-γ levels, as compared to the levels for the OVA/CAF09-immunized group, while the levels of IFN-γ were not significantly increased for OVA adjuvanted with CAF24a or CAF24c (Figure 2C). The Ab responses against OVA, measured as total IgG levels, were low for all groups (results not shown).

### CAF24a Rapidly Localizes in the dLNs Upon i.m. Administration

**Figure 3:**
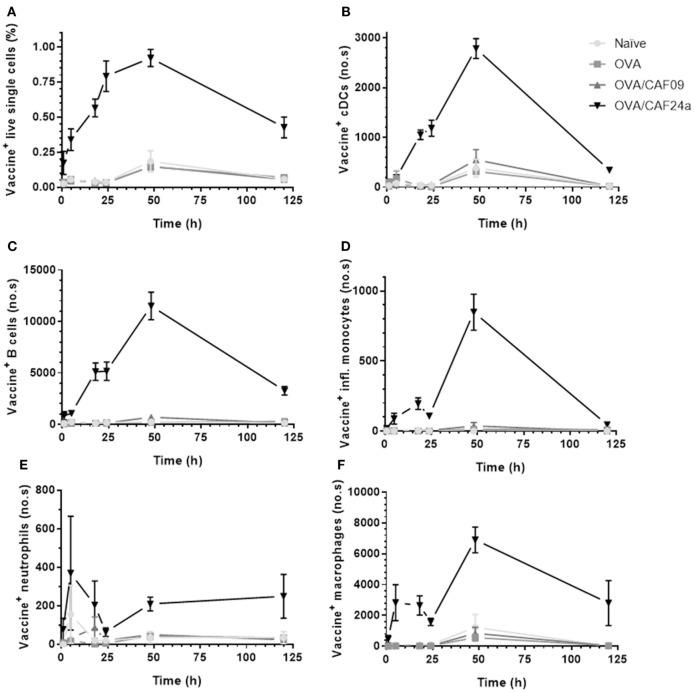
**(A)** Fraction of vaccine^+^ (adjuvant^+^, antigen^−/+^) lymphocytes, and number of **(B)** vaccine^+^ cDCs (CD11b^+^, CD11c^+^, MHC-II^+^), **(C)** vaccine^+^ B cells (CD19^+^), **(D)** vaccine^+^ inflammatory monocytes (CD11b^+^, Ly6C^+^), **(E)** vaccine^+^ neutrophils (Ly6G^+^), and **(F)** vaccine^+^ macrophages (CD11b^+^, F4/80^+^) in the draining lymph nodes following intramuscular immunization with ovalbumin (OVA) adjuvanted with cationic adjuvant formulation (CAF)09 and CAF24a, respectively, as compared to naïve mice and mice immunized with unadjuvanted OVA. *n* = 2 (naïve, unadjuvanted OVA) and *n* = 6 (OVA/CAF24a and OVA/CAF09), data points represent mean ± SEM.

**Figure 4:**
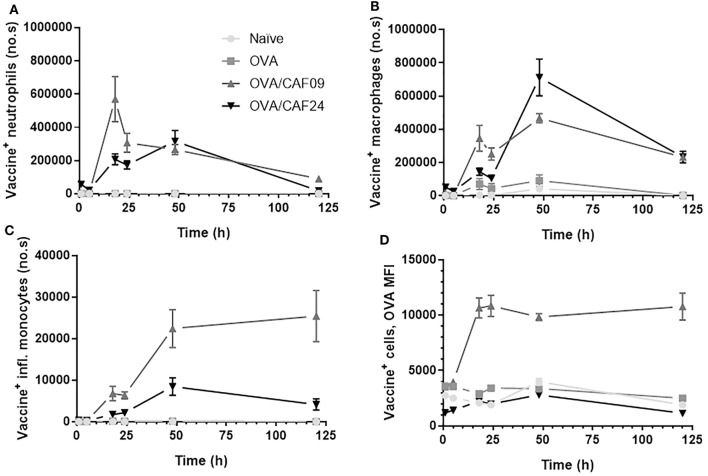
Number of **(A)** vaccine^+^ (adjuvant^+^antigen^+^) neutrophils, **(B)** vaccine^+^ macrophages, and **(C)** vaccine^+^ inflammatory monocytes in muscle following intramuscular administration of ovalbumin (OVA) adjuvanted with cationic adjuvant formulation (CAF)09 and CAF24a as compared to naïve mice and mice immunized with unadjuvanted OVA. **(D)** Mean fluorescence intensity (MFI) of OVA in vaccine^+^ muscle cells. *n* = 2 (naïve, unadjuvanted OVA) and *n* = 6 (OVA/CAF24a and OVA/CAF09). Data points represent mean ± SEM.

The difference in the immune responses induced with CAF24a and CAF09-adjuvanted OVA was investigated further by evaluating the distribution and cellular association in muscle and inguinal dLNs of fluorescently labeled AF647-OVA adjuvanted with DiO-CAF24a and DiO-CAF09, respectively, for 120 h following i.m. administration. We chose the i.m. immunization route for these studies because it enables us to evaluate influx of innate immune cells by excision of the SOI. The vaccine (adjuvant and antigen) localized rapidly in the dLNs upon i.m. immunization with OVA/CAF24a, whereas OVA/CAF09 remained at the SOI (Figure 3A) as previously reported (10). The CAF24a-adjuvanted vaccine was detected in the dLNs already 1 h after immunization, suggesting that either fast free drainage or transport mediated by draining cells account for the relocalization to the dLNs. At this time point, OVA/CAF24a was mainly associated with B cells and macrophages, whereas there were very few vaccine-positive inflammatory monocytes and neutrophils (Figure S5 in Supplementary Material). This contrasts the finding for the squalene emulsion-based adjuvant MF59^®^, that neutrophils and monocytes transport MF59^®^ and antigen from the SOI to the dLN (16). However, the lack of vaccine-positive neutrophils and inflammatory monocytes in the dLNs upon immunization with OVA/CAF24a might be a consequence of very rapid cell lysis *en route* or upon entry in the dLNs, which may eventually result in the release of unprocessed vaccine particles in the LNs (Figure S5 in Supplementary Material). At later time points, the rapid localization of OVA/CAF24a in the dLNs was accompanied by a simultaneous fast association of the vaccine with the cDCs (CD11b^+^, CD11c^+^, MHC-II^+^, Figure 3B). These include, among others, cross-priming CD8α^+^ and CD103^+^ DC subsets. The CAF24a-adjuvanted vaccine did also associate with a large number of B cells (CD19^+^). It is likely that there is no preferential association with specific cell subsets, but that the vaccine rather associates with a number of different types of immune cells found in the different LN compartments (Figure 3C). While cDCs and B cells were not recovered in the muscle in appreciable amounts (results not shown), vaccine association with inflammatory monocytes, macrophages, and neutrophils was detectable in both the muscle and dLNs (Figures 3D–F and 4A–C), indicating that transport of cell-associated vaccine from the SOI to the dLNs is mediated by one or several of these subsets. By contrast, OVA/CAF09 did not localize to the dLNs at detectable levels within the 120 h study period (Figure 3), indicating that CAF09 remains as a depot at the SOI within the duration of the study.

### CAF24a Associates With Immune Cells in Muscle 1 h After Administration, Whereas CAF09 Association to Immune Cells Is First Detectable After 18 h

At the SOI, we could detect OVA/CAF24a in neutrophils and macrophages shortly after immunization, while OVA/CAF09 association to neutrophils, macrophages, or inflammatory monocytes was first measured 18 h after administration (Figures 4A–C; Figure S6 in Supplementary Material). Thus, the rapid uptake of OVA/CAF24a by neutrophils and macrophages may account for the early localization of vaccine in the dLNs, although significant numbers of neutrophils could not be measured in the dLNs at this time point (Figure 3E). Interestingly, the fraction of OVA associated with each vaccine^+^ cell [measured as the mean fluorescence intensity (MFI)] was high in the OVA/CAF09-immunized group during the entire study period, while cells from mice immunized with OVA/CAF24a displayed only low levels of OVA throughout the study period (Figure 4D). This indicates that CAF09 forms a depot within the muscle cells at the SOI, as shown in previous studies with CAF09 and other cationic liposomal formulations (10–12). By contrast, a small fraction of OVA was retained at the SOI after immunization with OVA/CAF24a, although an appreciable number of cells was adjuvant positive (Figure 4). The relatively high OVA MFI-values measured in the OVA/CAF09-immunized mice, as compared to the OVA/CAF24a-immunized mice, may also be a result of aggregation of CAF09 upon addition of OVA, which has been observed in previous studies (10). The absolute values for the MFI of CAF09 and CAF24a cannot be directly compared because the specific compositions of the adjuvants affect the overall fluorescence intensity of the formulations.

### Immunization s.c With OVA/CAF24a Results in Induction of Significantly Stronger CTL Responses Than Immunization With OVA/CAF09

**Figure 5:**
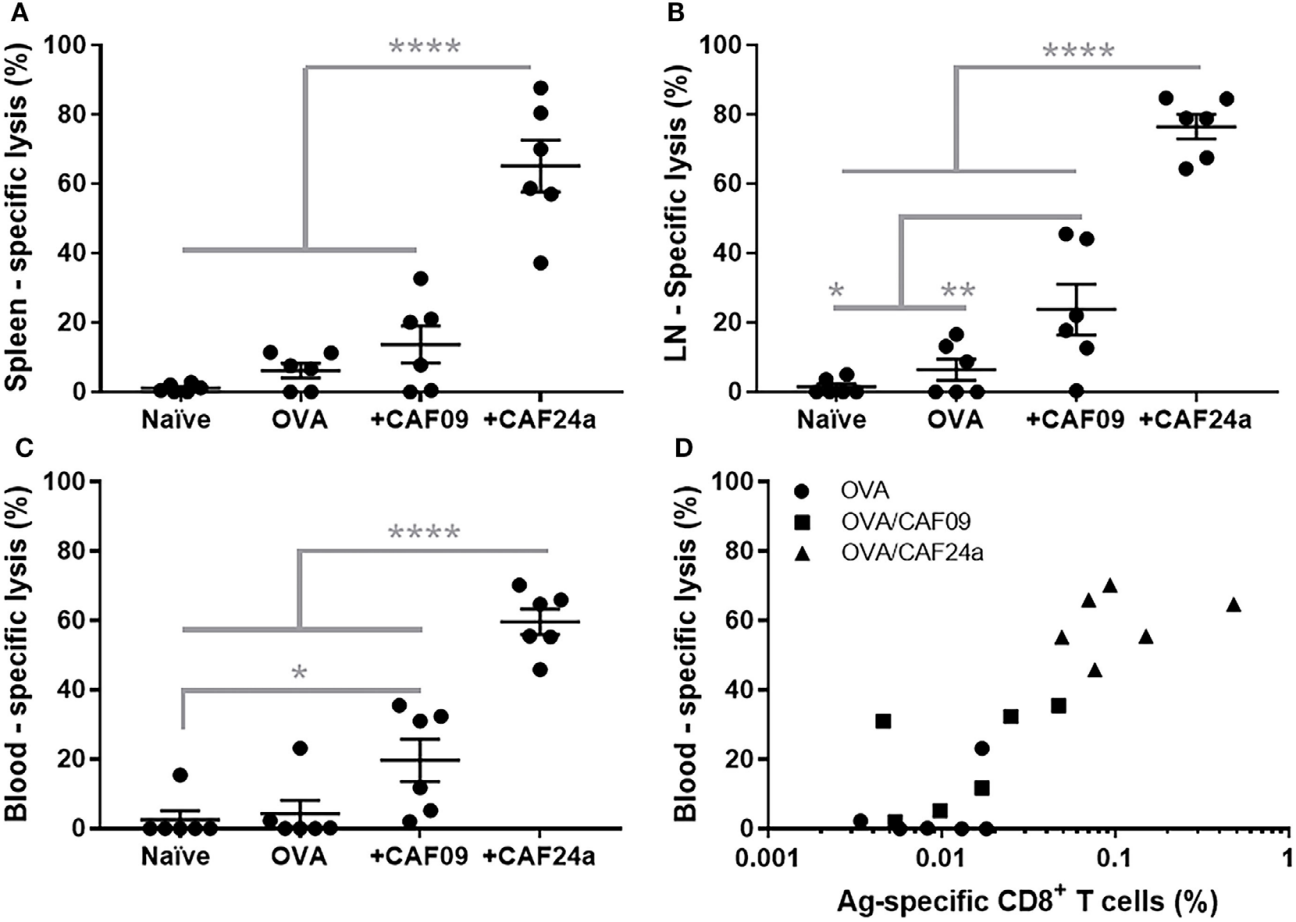
Specific lysis of SIINFEKL-loaded splenocytes 24 h after intravenous injection in **(A)** spleen, **(B)** draining lymph nodes, and **(C)** blood in naïve mice and following subcutaneous immunization with 1 μg/dose OVA, OVA/cationic adjuvant formulation (CAF)09, and OVA/CAF24a, respectively. **(D)** Comparison of the levels of SIINFEKL-specific CD8^+^ T cells and the specific lysis in the blood in individual mice. Correlation for the combined groups: Spearman, *p* = 0.0001. The results are from one of two repeated studies. *n* = 6, lines with bars represent mean ± SEM. *p ≤ 0.05, **p ≤ 0.01, ****p ≤ 0.0001, one-way ANOVA with Tukey’s post-test.

The effector function of the OVA-specific CD8^+^ T cells induced by immunization with CAF24a- and CAF09-adjuvanted OVA (OVA dose of 1 µg), respectively, was evaluated as the ratio between CFSE^hi^, SIINFEKL-pulsed splenocytes and CFSE^low^ non-pulsed splenocytes from naïve mice, which were recovered from immunized mice 24 h after i.v. injection of 1.6 × 10^6^ cells. A significantly higher antigen-specific lytic activity was measured for mice immunized with CAF24a-adjuvanted OVA, as compared to that for mice immunized with CAF09-adjuvanted OVA, in the spleen, dLNs, and blood following s.c. immunization (Figures 5A–C). The specific lysis following s.c. immunization with both adjuvants did not match the corresponding responses following i.p. immunization, which resulted in 100% specific lysis (results not shown).

To evaluate the effector function of the antigen-specific CD8^+^ T cells (capacity to lyse antigen-presenting target cells), a dose of 1 µg OVA was used, which resulted in lower levels of antigen-specific CD8^+^ T cells in the blood (Figure 5D) as compared to immunization with a dose of 10 µg OVA used in the immunization studies presented above (Figure 4B). However, there was a positive correlation (Spearman, *p* = 0.0001) between the percentage of antigen-specific CD8^+^ T cells and antigen-specific lysis (Figure 5D).

## Discussion

The present study provides proof-of-concept that nanoemulsions containing poly(I:C) induces antigen-specific CD8^+^ T-cell responses upon s.c. immunization, which appeared to have a significant antigen-specific lytic effector function in an *in vivo* assay. Thus, CAF24 might have potential as a CD8^+^ T-cell inducing adjuvant for i.m. and s.c. administration. The magnitudes of the immune responses induced by i.p. immunization with OVA adjuvanted with emulsions containing MMG and poly(I:C) (CAF24a) were similar to the magnitudes previously measured for the liposome-based CAF09 adjuvant (9, 10). This was expected, because immunization with a number of different antigens adjuvanted with CAF09 *via* the i.p. route has been shown to induce very robust and remarkably strong CD8^+^ T-cell responses (9). The magnitude of the CTL responses depends on the administration route, with the i.p. route being quantitatively superior to s.c./i.m. A likely explanation for this is that the number of targeted cross-priming DCs in the respective dLNs is highest following i.p. immunization due to increased free drainage of vaccine particles.

These results suggest that the adjuvant mechanisms of CAF09 and CAF24a might be very different, possibly as a result of differences in physicochemical properties, eventually being decisive for the biodistribution measured upon i.m. administration. Accumulation of MF59^®^-adjuvanted, fluorescently labeled antigen in dLN-resident macrophages and B cells has been observed 6 h following i.m. immunization (27). A comparable biodistribution profile was evident after i.m. immunization with a CAF24a-adjuvanted vaccine, which associated with lymphocytes and macrophages in the dLNs few hours after i.m. administration. By contrast, DDA-based liposomes have been shown to form persistent depots at the SOI (12). Upon i.m. immunization with CAF24a, a fraction of the vaccine also remained at the SOI throughout the entire study period. Thus, the formation of a nanoemulsion depot at the SOI does not prevent induction of functional CD8^+^ T-cell responses, possibly because it is the drainage kinetics of the vaccine to the dLNs that is rate limiting for the induction of CTL responses. The two w/o emulsion adjuvants incomplete Freund’s adjuvant and Montanide ISA-51, both in combination with a TLR9-ligand, were shown to induce significant antigen-specific CD8^+^ T-cell responses in clinical trials (28, 29). The w/o emulsions form persistent depots at the SOI (30, 31), indicating that emulsion adjuvants can be engineered to have CTL-inducing abilities. However, a drawback is that the antigen remaining at the SOI may attract activated antigen-specific CD8^+^ T cells, resulting in systemic depletion of antigen-specific CTLs (30). Furthermore, moderate and serious adverse events have been reported in several clinical trials with Montanide ISA-51 (32).

Based on the physicochemical properties of the liposomal CAF09 and the emulsion-based CAF24a, differences in draining patterns are expected. CAF09, which is strongly cationic, is hypothesized to interact rapidly with negatively charged interstitial proteins upon administration, as previously observed for DDA:trehalose-6,6′-dibehenate-liposomes (11). By contrast, the emulsion droplets of CAF24a were found to have a reduced zeta-potential as compared to CAF09, which might be explained by the presence of the surfactants Tween^®^ 60 and Span^®^ 60, which shield the charge of the cationic headgroups. Furthermore, Tween^®^ 60 contains three polyethylene glycol moieties, which to some degree may prevent aggregation with interstitial proteins. If aggregation of the emulsion droplets upon administration is prevented, some degree of free drainage to the dLNs may be possible, because the particle size is below approximately 200 nm, which is suggested to be a prerequisite for entering the lymphatics through the epithelial cell lining of the lymphatic ducts (14, 33). Further studies are needed to evaluate the interaction with interstitial proteins and the kinetics of antigen/adjuvant processing in order to understand the mechanism(s) of antigen association to the adjuvant *in vivo*.

Both CAF09 and CAF24a associated with neutrophils, macrophages, and inflammatory monocytes at different levels upon i.m. administration. Immunization with MF59^®^ also resulted in an influx of neutrophils, monocytes, eosinophils, macrophages, and mDCs at the SOI (16). In a study evaluating the efficacy of a soy bean oil with ginseng root saponin-emulsion as a vaccine adjuvant, the significantly increased cytokine levels in the muscle SOI 3 h after immunization indicates that macrophages are recruited to the SOI (34). Hence, the environment at the SOI is dominated by influx of immune cells, which is an expected response to the immunostimulatory effects of the adjuvants. Thus, the cellular transport and/or free drainage of the emulsion-based adjuvant may be rate limiting for the presentation of the vaccine to cross-priming DCs. Therefore, transport of intact vaccine particles by neutrophils and monocytes might play a role. Thus, MF59^®^ is localized in the dLNs in association with neutrophils and monocytes a few hours after immunization (16), as shown here for CAF24a. Furthermore, intradermally (i.d.) administered *Mycobacterium bovis* bacilli Calmette-Guérin was transported to the dLNs by neutrophils entering the SOI (35). The transport function by neutrophils might be dependent on the particles or pathogens in question, as i.d. administered herpes simplex virus type 1 was not transported by neutrophils to the draining lymph nodes (dLNs), despite neutrophils entering the SOI (36). These results indicate that particles with specific physicochemical properties are transported to the dLNs by cell-mediated transport, which in turn might increase the propensity for targeting and activating LN-resident cross-priming DCs.

The antigen-specific lytic response induced by immunization with CAF24a correlates with the recovery of the vaccine in the dLNs, particularly the fraction of vaccine associated with cDCs. This indicates that targeting of LN-resident cross-priming DCs increases the chances of inducing a robust CD8^+^ T-cell response. However, targeting of other cells in the LNs might also play a role in the induction of CTL responses, e.g., plasmacytoid DCs, which are suggested to promote cross-priming facilitated by cross-presenting DCs *via* expression of type I IFNs (37).

## Conclusion

Incorporation of DDA, MMG-1, and poly(I:C) into a squalane-based nanoemulsion (CAF24a) resulted in a positively charged emulsion with an average droplet diameter below 200 nm. Strong antigen-specific CTL responses were induced upon s.c. immunization with the emulsion, which correlated with the induction of strong antigen-specific lytic responses. These responses were significantly higher than the responses induced by the liposome-based adjuvant, CAF09, which is composed of the same immunostimulatory compounds, indicating that the specific type of delivery system and its pharmacokinetics and pharmacodynamics *in vivo* play important roles for induction of CTLs after s.c. administration. Thus, CAF24a is a promising candidate adjuvant for vaccines aiming to elicit high CTL responses to prevent or treat infectious diseases, because it can be administered *via* the clinically acceptable s.c. and i.m. routes.

## Ethics Statement

All animal experiments were carried out in accordance with the national Danish guidelines for animal experiments and EU directive 2010/63/EU for animal experiments, as approved by the Danish Animal Experiments Inspectorate (license number 2014-15-2934-01065). All procedures were refined to provide maximal comfort and minimal stress for the animals.

## Author Contributions

SS, GP, KK, CF, and DC designed the study. SS, GP, and MN prepared the laboratory work and analyzed the data. SS, GP, MN, CF, and DC interpreted the data. SS, GP, CF, and DC drafted the manuscript. SS, GP, KK, TR, PA, CF, and DC provided scientific input throughout the study period and draft of the manuscript.

## Conflict of Interest Statement

SS, GP, KK, PA, and DC are employed by Statens Serum Institut, which is a nonprofit government research facility, holding patents on the cationic adjuvant formulations (CAF).

## Abbreviations

Ab, antibody; CAF, cationic adjuvant formulation; CFSE, carboxyfluorescein succinimidyl ester; cryo-TEM, cryo-transmission electron microscopy; CTL, cytotoxic T-lymphocyte; DC, dendritic cell; DDA, dimethyldioctadecylammonium; DiO, 3,3′-dioctadecyloxacarbocyanine perchlorate; dLN, draining lymph node; DSPE, distearoylphosphoethanolamine; ELISA, enzyme-linked immunosorbent assay; HSM, high shear mixing; i.d., intradermal; IFN, interferon; i.m., intramuscular; i.p., intraperitoneal; i.v., intravenous; LN, lymph node; MHC, major histocompatibility complex; MMG, monomycoloyl glycerol; o/w, oil-in-water; PBS, phosphate-buffered saline; PDI, polydispersity index; poly(I:C), polyinosinic:polycytidylic acid; s.c., subcutaneous; SOI, site of injection; TDB, trehalose-6,6′-dibehenate; TLR, toll-like receptor; w/o, water-in-oil; Z-average, intensity-weighted average hydrodynamic diameter.
