# Supplementary material for: Induction of Cytotoxic T-Lymphocyte Responses Upon Subcutaneous Administration of a Subunit Vaccine Adjuvanted With an Emulsion Containing the Toll-Like Receptor 3 Ligand Poly(I:C)
Source: Front Immunol. 2018 Apr 30;9:898. doi: 10.3389/fimmu.2018.00898 (PMC5936752; doi:10.3389/fimmu.2018.00898)
Supplement: Supplementary file 1 [file Data_Sheet_1.docx]

*Supplementary material*

Induction of cytotoxic T-lymphocyte responses upon subcutaneous administration of a subunit vaccine adjuvanted with an emulsion containing the TLR3 ligand poly(I:C)

Signe Tandrup Schmidt^1,2^, Gabriel Kristian Pedersen^2^, Malene Aaby Neustrup^1^, Karen Smith Korsholm^2^, Thomas Rades^1^, Peter Andersen^2^, Camilla Foged^1,#^, and Dennis Christensen^2,#,*^

^1^ Department of Pharmacy, Faculty of Health and Medical Sciences, University of Copenhagen, Copenhagen, Denmark

^2^ Department of Infectious Disease Immunology, Statens Serum Institut, Copenhagen, Denmark

# Shared senior authorship

***** Correspondence: [den@ssi.dk](mailto:den@ssi.dk). tel. +45 3268 3804


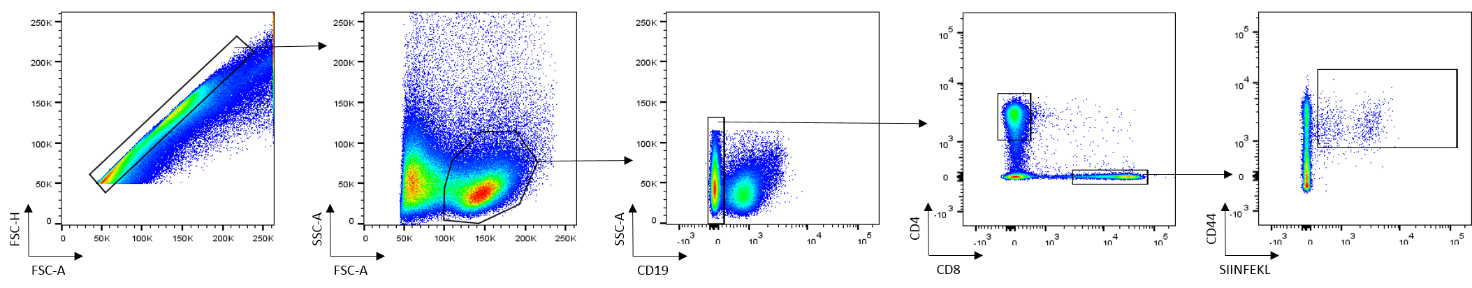


**Figure S1:** Gating strategy for defining SIINFEKL-specific CD44^+^ CD8^+^ T cells in the blood of immunized mice. In the example, OVA/CAF24a was administered.


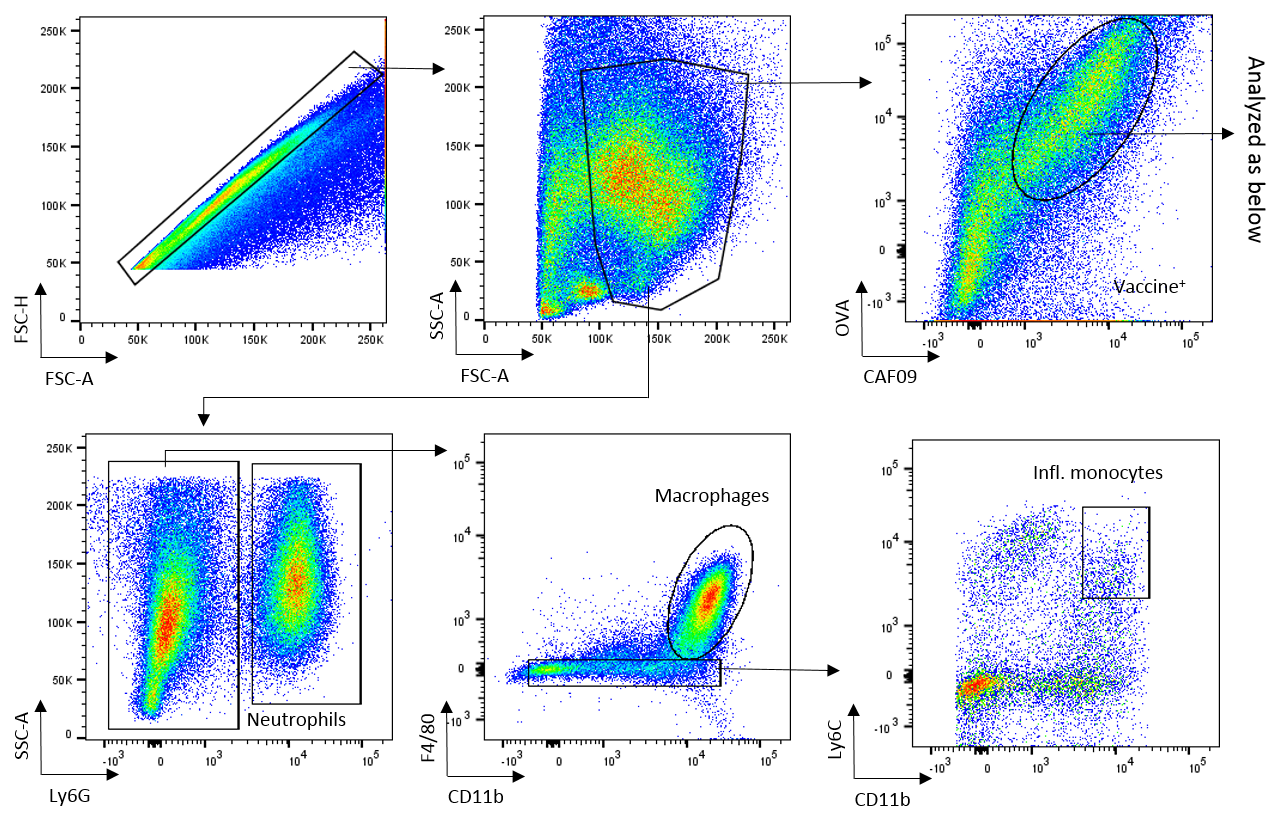


**Figure S2:** Gating strategy for defining immune cell subsets in the muscle. The example is 18 h after OVA/CAF09 administration.


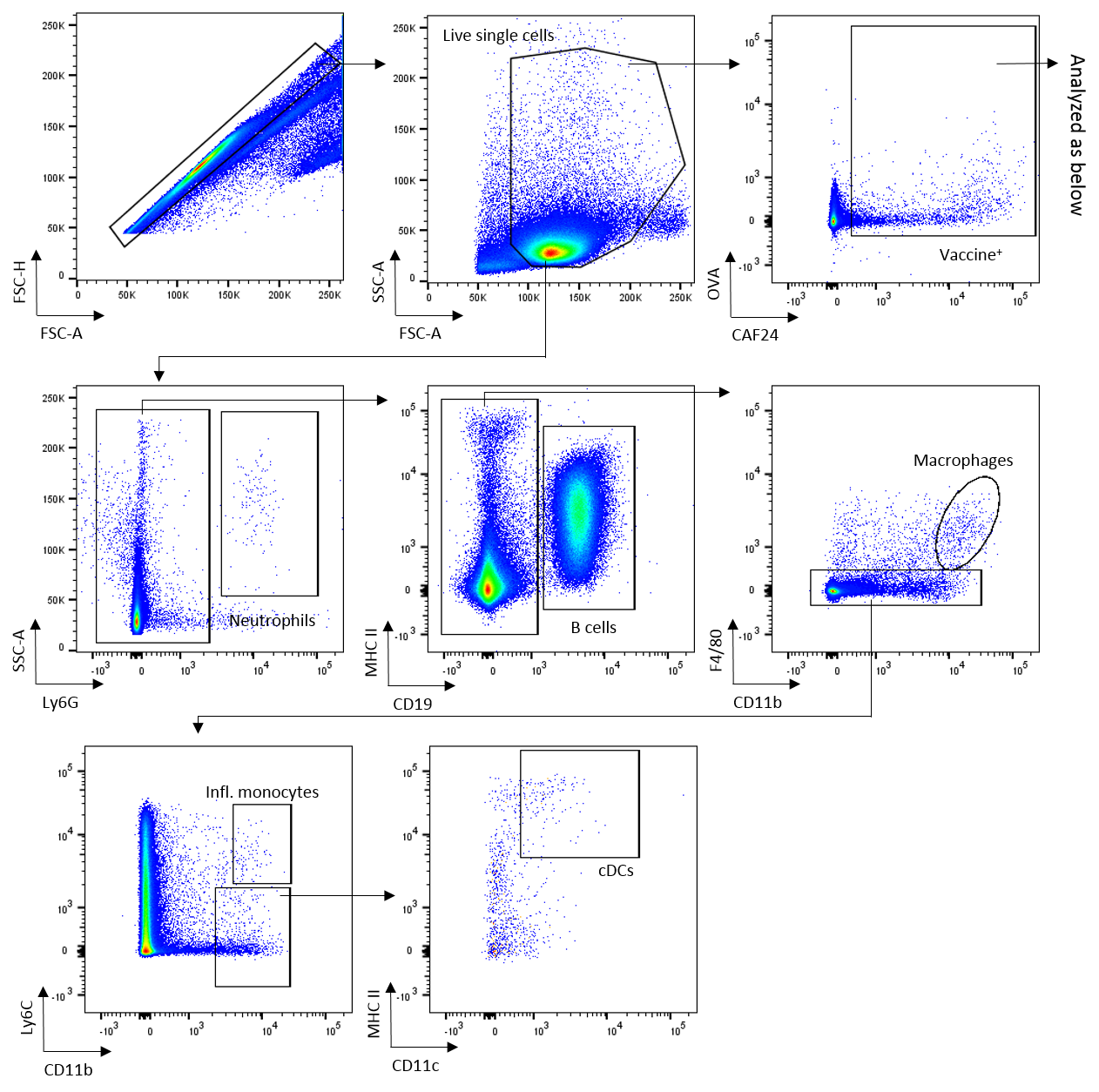


**Figure S3:** Gating strategy for defining immune cell subsets in the dLNs. The example is 24 h after OVA/CAF24a administration.


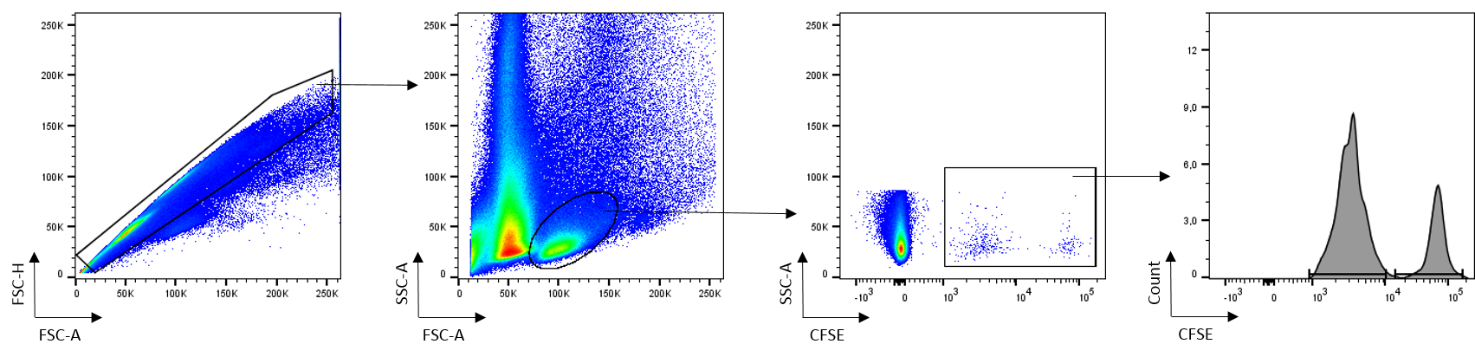


**Figure S4:** Gating strategy for defining transferred, CFSE-pulsed splenocytes in the blood of immunized mice. In the example, OVA/CAF24a was administered.

**Figure S5:** The number of vaccine^+^ cells within different subsets in the dLNs at 1 h after immunization. n=2 (naïve, unadjuvanted OVA) and 6 (OVA/CAF24a and OVA/CAF09), mean±SEM.

**Figure S6:** The number of vaccine^+^ cells within different subsets in the muscles at 1 h after immunization. n=2 (naïve, unadjuvanted OVA) and 6 (OVA/CAF24a and OVA/CAF09), mean±SEM.
